# Structural and functional insights into cellulosomes: masters of plant cell wall degradation

**DOI:** 10.3389/fmicb.2025.1638551

**Published:** 2025-09-17

**Authors:** Nataša Lindič, Maša Vodovnik

**Affiliations:** ^1^Department of Biochemistry, Molecular and Structural Biology, Jožef Stefan Institute, Ljubljana, Slovenia; ^2^Department of Microbiology, Biotechnical Faculty, University of Ljubljana, Ljubljana, Slovenia

**Keywords:** cellulosome, cellulose, cellulase, CBM, cohesin, dockerin, scaffoldin, SLH

## Abstract

Cellulosomes are complex multi-enzyme systems that enable efficient cellulose breakdown in some anaerobic bacteria and fungi. Understanding cellulosome functionality plays a crucial role in expanding their potential for industrial plant biomass degradation and valorization. While knowledge on these intricate structures has been accumulating for several decades, recent insights into their modular architecture, dynamic assembly mechanisms, and potential for synthetic biology-driven redesign for biotechnological applications call for a comprehensive re-evaluation of their structural and functional complexity. This review explores recent advances in understanding these cellulolytic nanomachines, focusing on substrate recognition and binding mechanisms, including the roles of carbohydrate-binding modules and cohesin-dockerin interactions. Cell-surface mechanisms that allow these complexes to attach to and effectively degrade plant biomass are also reviewed. Furthermore, structural adaptations to diverse substrates and environmental conditions are discussed, highlighting the flexibility and the interplay between the cellulosomal components, both catalytic and non-catalytic, and their impact on optimizing cellulose degradation, including carbon source sensing, and its role in modulating cellulosome architecture and activity.

## 1 Introduction

Degradation of cellulose, the most abundant organic polymer on Earth, is instrumental in the global carbon cycle. Cellulosomes are large multienzyme complexes that break down cellulose-rich plant material. They are found in natural environments like soil, compost, and the rumen of herbivores, as well as in artificial systems such as anaerobic digesters. They are primarily produced by anaerobic bacteria, although similar structures have also been detected in some anaerobic fungi (Artzi et al., 2017; Lillington et al., 2021; Minor et al., 2024; Vodovnik and Lindič, 2025). In a recent genomic study involving the analysis of 305,693 bacterial genomes, 33 bacterial species with the genomic capacity to produce cellulosomes (including 10 previously unreported) were identified. This analysis revealed that cellulosome-producing capacity originates mostly from four bacterial genera: *Acetivibrio*, *Ruminococcus*, *Ruminiclostridium* and *Clostridium* (Minor et al., 2024). Cellulosomal complexes have been shown to enhance biomass degradation by optimizing enzyme synergy and substrate targeting. This offers an interesting option for different industrial applications, particularly second-generation biofuels. Furthermore, the modular architecture of cellulosomes has inspired the design of artificial complexes [designer cellulosomes (DCs)], tailored for even more efficient and targeted degradation of plant biomass (Vodovnik and Lindič, 2025).

**Figure 1:**
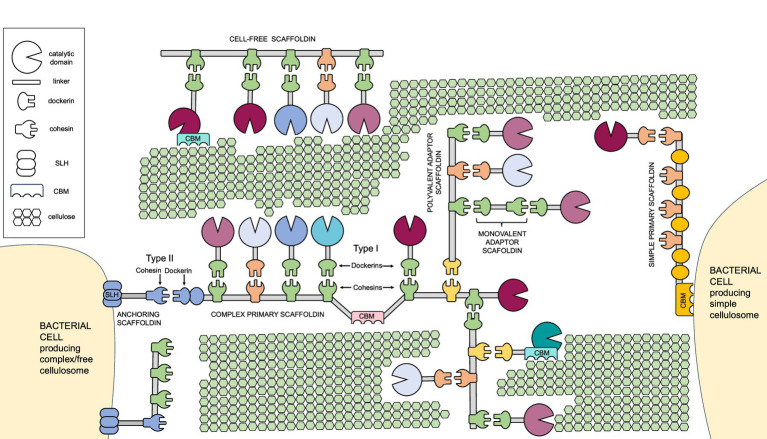
The architectural diversity of the cellulosomes. Major structural components of different types of cellulosomes and their attachment to the host cells are shown. CBM, carbohydrate binding module; SLH, S-layer homology domain.

Cellulosomes are architecturally versatile complexes (Figure 1) that play a pivotal role in the process of cellulose degradation by integrating carbohydrate-degrading enzymes onto a flexible structural backbone - the scaffoldin. Scaffoldins are large non-catalytic proteins that organize and anchor catalytic components via multiple cohesin domains. Cohesin domains interact specifically and strongly with the dockerin domains of catalytic subunits. Catalytic domains typically include cellulases, hemicellulases, pectinases, and other carbohydrate-active enzymes (reviewed in Artzi et al., 2017). Additionally, accessory proteins such as expansin-type proteins (Chen C, et al., 2016; Cosgrove, 2017), proteases (Levy-Assaraf et al., 2013) and protease inhibitors (Ó Cuív et al., 2013) are also present. Interestingly, proteins with CotH-like domains have also been identified within some cellulosomes (Vodovnik et al., 2013). Although their role is not clear yet, findings suggest that CotH proteins in cellulosomes may serve in two ways: as structural components facilitating the assembly of the complex as well as regulatory elements via their kinase activity (Ben David et al., 2015; Blouzard et al., 2010; Lillington et al., 2023; Zverlov et al., 2003). Scaffoldins form the structural backbone of the cellulosomes. The most common scaffoldins, called primary scaffoldins, usually have multiple cohesin domains that bind dockerin-tagged enzymes. Most scaffoldins also have a special dockerin that helps attach them to the cell surface by binding to cohesins on anchoring scaffoldins. These protein connect the complex to the cell surface either non-covalently via S-layer homology (SLH) domains or covalently through sortase-mediated attachment. In more complex cellulosomes, adaptor scaffoldins have also been found, linking two scaffoldins or a scaffoldin and an enzyme (reviewed in Artzi et al., 2017, 2014). Cellulosomes also work as a link between the bacterial cell and the substrate. The attachment is mediated via scaffoldin’s carbohydrate-binding modules (CBMs) and results in concentrating enzymes directly on the substrate, additionally enhancing its degradation efficiency (Galera-Prat et al., 2018; Valbuena et al., 2009; Verdorfer et al., 2017). The cellulosome-mediated sensing and degradation of cellulose biomass is a result of several complex mechanisms, including substrate recognition and binding, structural adaptation, cell-surface anchoring, enzyme synergy and proximity effects. Recent advances in studying these mechanisms with emphasis on functional and structural aspects of cellulosomes are reviewed below.

## 2 Carbohydrate-binding modules and their interplay with other cellulosomal domains

**Figure 2:**
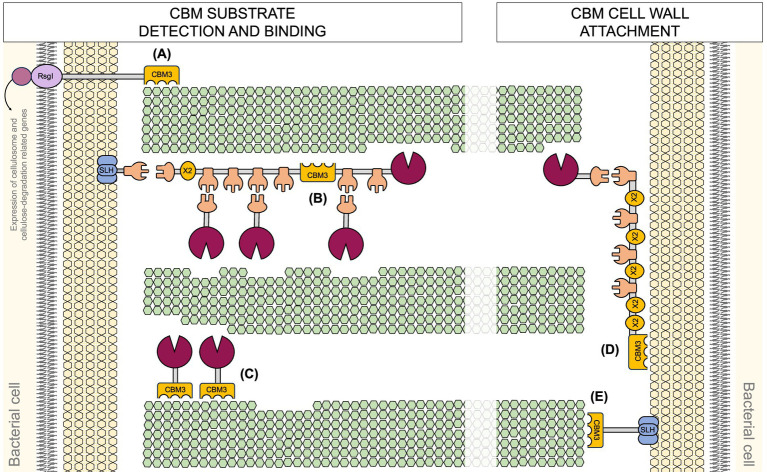
Diverse (proposed) roles of CBM3 domains in cellulolytic bacteria. **(A)** Carbohydrate sensing. **(B)** Cellulosome-substrate bridging. **(C)** Free cellulase activity enhancement. **(D)** Simple cellulosome-host interactions. **(E)** Cell attachment to the substrate.

Carbohydrate binding modules (CBMs) are non-catalytic domains found on cellulosomal scaffoldins as well as various carbohydrate-active enzymes (CAZymes). CBMs play diverse roles in cellulolytic bacteria (Figure 2). One of the crucial ones is substrate recognition, i.e., binding of the cellulosomal complex to specific carbohydrate structures within the plant biomass. CBMs are an integral part of the enzymes in structure and function. As such, they have also been shown to improve the thermostability of the enzymes (De Chellis et al., 2024b; Li et al., 2023), and disrupt the substrate to facilitate attack by the catalytic domain or other enzymes (Arantes and Saddler, 2010; Li and Liu, 2024; Reinikainen et al., 1992; You et al., 2024). In addition, CBMs in simple cellulosomes were proposed to be involved in cell-surface anchorage, described in section 3 in more detail. Furthermore, non-cellulosomal CBMs in cellulolytic bacteria have been shown to be a part of the unique carbohydrate-biosensing system. Herein, an extracellular CBM as a part of an anti-sigma factor RsgI was shown to detect polysaccharides. This leads to signal transduction and subsequently activation of gene transcription of cellulosomal systems and cellulase-related genes, potentially enabling a dynamic cellulosome assembly for efficient polysaccharide hydrolysis (Chen C, et al., 2023; Dong et al., 2024; Hershko Rimon et al., 2021; Kahel-Raifer et al., 2010; Sand et al., 2015; Yaniv et al., 2014).

### 2.1 CBM types

The diversity of CBM structures allows cellulosomes and CAZymes for a more specific substate recognition within heterogeneous plant biomass. Based on their tertiary structure/function similarities, particularly their ligand-binding sites, CBMs are classified into three types, type A, B and C. Type-A CBMs have a flat or platform-like binding site composed of aromatic residues that recognize the surface of crystalline polysaccharides (surface-binding CBMs). Type-B CBMs have a groove or cleft-like binding that can bind internally to single polysaccharide chains (endo-type CBMs). Type-C CBMs lack the extended binding-site grooves of type B CBMs and bind the shorter substrates at polysaccharide termini (exo-type; Boraston et al., 2004; Creagh et al., 1996; Gilbert et al., 2013; Liu et al., 2022). Recently, a novel ligand preference-based classification has been proposed, describing four types of CBMs. Herein, type I and II include CBMs specific for non-branched carbohydrates, while type III and IV CBMs recognize and attach to branched polysaccharides and enhance the hydrolysis of substrates containing side chains (Liu et al., 2021).

### 2.2 CBM3 structure

According to their primary structure, CBMs are currently classified into 106 families (Carbohydrate Active Enzymes Database 2025;^1^ Boraston et al., 2004; Creagh et al., 1996; Drula et al., 2022; Gilbert et al., 2013; Liu et al., 2022; You et al., 2024). Among these, CBM3 modules (Figure 2) are particularly frequently found in cellulosomes. CBM3a are known for strong binding to crystalline cellulose, which proved essential for its degradation (Yaniv et al., 2012; Yaniv et al., 2011). CBM3s are approximately 150 amino acids long. They typically adopt a *β*-jelly roll fold with nine β-strands forming one flat and one curved β-sheet. While the role of the concave surface remains unclear (Hershko Rimon et al., 2021; Yaniv et al., 2012), the flat surface composed of aromatic residues (His, Trp, Tyr and an Arg-Asp ion pair) in a linear strip aligns with the glucose rings of the cellulose chain (Ding et al., 2006; Tormo et al., 1996). Mutations herein have been shown to affect substrate specificity. Single mutations can result in the loss of binding to cellulose (Cai et al., 2011), restoration of binding (Yaniv et al., 2012) or altered adhesion properties (von Schantz et al., 2012). In a non-cellulosomal CBM3 from *Clostridium thermocellum (C. thermocellum)*, proposed to serve as a surface-anchored polysaccharide biosensor, the lack of the required number of key aromatic residues might explain the preference for xylan binding (Hershko Rimon et al., 2021). Furthermore, multimodal binding of the CBM to the substrate was revealed using recently developed acoustic force spectroscopy-based methods (Chundawat et al., 2024; De Chellis et al., 2024a; Hackl et al., 2022). Specifically, the pulling on the wild-type CBM3a and consequently its dissociation from the fiber revealed that the planar aromatic binding motif contains two carbohydrate binding regions (Hackl et al., 2022).

In addition to multimodal binding CBM characteristics, multivalent CBMs have been discovered. These exhibit dual-specificity binding of both, cellulose and xyloglucan, which is influenced by substrate cleft topology (Hernandez-Gomez et al., 2015; Levi Hevroni et al., 2020; Venditto et al., 2016). Notably, certain multivalent CBMs, such as recently discovered family 92, feature unique three-site binding modes that were proposed to facilitate polysaccharide cross-linking in nature (Hao et al., 2024). In contrast to CBMs discussed so far that are functionally and structurally autonomous from their associated catalytic domains, there are some exceptions like group c CBM3s (CBM3cs). CBM3cs are integral to the substrate-binding clefts of glycoside hydrolases 9 (GH9) and lack conserved ligand-binding residues. Thus, they do not bind cellulose directly. Instead, CBM3cs play a structural or catalytic-assisting role. Here, aromatic residues are replaced with polar amino acids, that disrupt cellulose inter-chain hydrogen bonds, leading the cellulose chains into the catalytic domain’s active site for hydrolysis (Gilad et al., 2003; Jindou et al., 2006; Kuch et al., 2023; Li et al., 2010; Sakon et al., 1997). All in all, since the substrate specificity of CBMs is intricately tied to critical residues and their spatial structure, targeted modification of these residues is a promising strategy for directed evolution and diversification of these modules (Zhou et al., 2024).

### 2.3 X2 modules

All simple primary scaffoldins found in *C. cellulovorans, Ruminiclostridium cellulolyticum (R. cellulolyticum), Clostridium josui* and *Clostridium saccharoperbutylacetonicum* (Dassa et al., 2017; Levi Hevroni et al., 2020) contain X2 modules. X2 modules are hydrophilic domains of approximately 100 amino acids (Mosbah et al., 2000) widely distributed among cellulosome-producing bacteria (Dassa et al., 2017; Doi et al., 1994; Lamed and Bayer, 1988) and free cellulases (Pasari et al., 2017; Ravachol et al., 2015; Vita et al., 2019). The function of X2 modules remains unclear, though they have been proposed to contribute to cellulose binding and hydrolysis (Aburaya et al., 2015; Pasari et al., 2017; Tamaru et al., 2011), to stabilize adjacent cohesin domains (Levi Hevroni et al., 2020) or interact with bacterial cell walls (Kosugi et al., 2004; Tarraran et al., 2021). Nevertheless, direct binding of a cell wall component has never been experimentally demonstrated. Moreover, recent studies demonstrate that the X2 modules within the CipC scaffoldin lack direct binding affinity to both, the bacterial cell surface and cellulose (Tao et al., 2022). The structural analysis of the X2 module shows a predominantly hydrophilic surface with a shallow hydrophobic groove (Mosbah et al., 2000) which might explain the lack of direct cell wall binding. When the loop-located motif (NGNT), conserved in all X2 modules, is removed, this groove widens (Tao et al., 2022) into conformation that resembles CBM structures (Luís et al., 2013; Pasari et al., 2017) and presumably allows weak cell wall interactions (Tao et al., 2022). This motif was found to be crucial for cellulose hydrolysis in *Clostridium cellulolyticum (C. cellulolyticum)* (Tao et al., 2022). All in all, the X2 modules are located next to the CBM3a modules in free cellulases and the CBM3-X2 module has shown a better binding affinity to crystalline cellulose compared to CBM3 alone (Pasari et al., 2017; Zhang et al., 2018). Thus, the mechanism was proposed where X2 module interacts with CBM3a to promote its binding function (Tao et al., 2022). These findings offer a compelling framework for further investigations.

### 2.4 CBM-based engineering applications

CBMs have long been known to enhance enzymatic activity of their partner catalytic domains by ensuring their proximity to the substrate. In DCs, CBMs with different specificities have been used to direct complexes to different substrates (Lamote et al., 2023; Vanderstraeten et al., 2022a). Fusing various *Ruminoclostridium thermocellum* CBMs with specific binding activities to multifunctional cellulosomal enzyme CelE enabled its activity on diverse substrates like lichenan, xylan, and mannan (Walker et al., 2015). Similarly, CBM binding specificity affected the potency of chimeric endoglucanases to degrade milled lignocellulosic materials (Ichikawa et al., 2016). In *Paenibacillus polymyxa* A18, the *X2-CBM3 module* in association with xyloglucanase and endoglucanase enhanced enzyme activity up to 4.6-fold (Pasari et al., 2017). Similar increase in enzymatic activity was observed for *Acetivibrio thermocellus* CBM3 *fused to GH5* endoglucanase from *Trichoderma viride* (Ran et al., 2024). Furthermore, CBM1 fused with lytic polysaccharide monooxygenases from *Neurospora crassa* enhanced fiber oxidation (Støpamo et al., 2024). Using CBMs with cutinase even increased PET plastic degradation, showcasing the potential of CBMs in developing environmentally friendly recycling strategies (Chen Y, et al., 2023). Surprisingly, reducing CBM-binding affinity by mutagenesis of aromatic residues in the planar binding motif while maintaining binding sites conformation can enhance the catalytic activity of CelE–CBM mutants on some cellulose substrates. This supports the hypothesis that non-productive binding hinders efficiency (Nemmaru et al., 2021) and provides additional confirmation that the optimal binding strength is critical for catalysis (Kari et al., 2018), as observed in studies on cellulases and expansins (Hepler and Cosgrove, 2019). The surface-charged interactions between slightly negative lignin and CAZymes were recently explored. Introducing and fine-tunning mutations to supercharge the surface of CBM and/or its endocellulase partners has been shown to enhance endocellulase thermostability, binding, and activity on cellulosic biomass (De Chellis et al., 2024b). This is in line with the observation that electrostatic interactions are involved in the binding of CBM3s to hemicellulose and that the xylan-binding ability of the CBM3s is affected by the ionic strength of the environment (Hershko Rimon et al., 2021). Altogether, these findings highlight the critical need to further investigate the complex interplay between the CBMs and their catalytic domain partners.

Incorporation or removal of non-catalytic domains such as CBMs from the polypeptide can modulate thermostability, binding specificity, and catalytic efficiency of the enzymatic module (Hettle et al., 2017; Kuch et al., 2023; Nakamura et al., 2016; Sammond et al., 2012; Tang et al., 2013; Walker et al., 2015). While most cellulase-associated CBMs are located N- or C-terminally, insertion of the CBM sequence within the sequence of the catalytic domain is less common. So far, it was identified in only a few xylanases (Flint et al., 1997; Wu et al., 2011). Additional example identified recently is the endoglucanase from thermophilic bacterium *Meiothermus taiwanensis* WR-220 (Ye et al., 2022). Structural analysis showed that this GH5-family endo-*β*-1,4-glucanase has a bipartite architecture featuring a Cel5A-like domain with an inserted CBM29-like domain. Deletion of this CBM domain significantly reduced the activity of the enzyme, while its insertion into the *Thermotoga maritima* Cel5A significantly enhanced affinity of the chimera to longer polysaccharides (Ye et al., 2022). Furthermore, superimposition of MtGlu5 with homologs and substrate-binding modeling suggest that the polysaccharide chain interacts with a continuous groove from the catalytic domain to the CBM. Mutagenesis experiments showed six tryptophans (three in the CBM and three in the catalytic domain) that are crucial for sugar binding. These findings highlight the synergistic effect of a CBM insertion into a GH domain of the enzyme and its potential for extending the substrate binding groove to increase affinity to longer substrates (Ye et al., 2022).

In a recent study by Vita et al. (2019), the specific features required to generate an efficient free or cellulosomal family-9 cellulase were identified. Specifically, removing the two C-terminal X2 modules and the CBM3b moiety from free-state Cel9A reduced its activity on crystalline cellulose. In contrast, adding these elements to cellulosomal Cel9G resulted in increased activity on crystalline cellulose (Vita et al., 2019). X2 and CBM3 together were demonstrated to be highly efficient in enhancing the activity of GHs toward the insoluble substrate (Pasari et al., 2017). Furthermore, in a DC system, the influence of CBM copy number within the scaffoldin (constructs containing two, one, or no CBMs) was evaluated. The results demonstrated that increasing the number of CBMs significantly enhances cellulosome efficiency by improving substrate binding. The construct with two CBMs achieved the highest sugar release from Avicel. A less pronounced effect was observed during the degradation of PASC (Anandharaj et al., 2020). All in all, tandem CBMs can coordinate catalytic domains by leveraging differences in binding affinity, ligand preference, and spatial arrangement of multiple enzymes into a single multimodular unit for complex substrate degradation (Kuch et al., 2023; Liu et al., 2024).

Variability in glycan recognition was also suggested to be an important aspect of efficient polysaccharide degradation and regulation of the transcription of cellulosomal proteins in response to different polysaccharides. For example, in the CBM-ome of *Ruminococcus flavefaciens* (*R. flavefaciens*), six novel CBM families binding β-glucans, β-mannans, and the pectic polysaccharide homogalacturonan were identified (Venditto et al., 2016). Similarly, in (*Pseudo*)*bacteroides cellulosolvens*, which was reported to contain the most complex known cellulosome system to date, a novel type B CBM has been shown to displays similarities to fibronectin type III (Fn3) domain and acts as an extracellular substrate biosensor with broad polysaccharide binding profile. This module exhibited extensive binding to chitosan and arabinoxylan, medium binding to amorphous cellulose and xylan, and weak binding to Avicel (Dong et al., 2024). Recent genomic and proteomic studies further emphasizes the variability of CBMs across different species (Dassa et al., 2017; Minor et al., 2024; Zhivin-Nissan et al., 2019).

Altogether, CBMs within cellulosomes and those in free CAZymes play essential roles in substrate targeting, enzymatic efficiency, and stability. Among them, cellulosome-prevalent CBM3a is well known for its strong affinity to crystalline cellulose, while X2 modules are thought to support CBM function and/or scaffoldin stability. Recent advances reveal additional layers of CBM functionality, including multimodal binding, dual substrate specificity, and roles in carbohydrate sensing and transcriptional regulation. From an engineering standpoint, CBMs serve as modifiable elements for tailoring enzymes and DCs with improved binding, activity, and specificity.

## 3 Cohesin-dockerin interactions

The primary interaction within the cellulosomal subunits is the interaction between dockerin and cohesin domains (Artzi et al., 2017; Bule et al., 2018a; Bule et al., 2017). It resembles a plug-and-socket mechanism, and can be classified based on their origin, specificity and structural features. According to their primary sequence, three types of cohesin-dockerin pairs have so far been identified: type I, type II, and type III. Type I pairs are commonly involved in enzyme organization within the cellulosome, while type II pairs facilitate cellulosome attachment to the cell surface (Artzi et al., 2017; Pinheiro et al., 2009). Exceptions where the situation is reversed have also been described. Namely, in *Bacteroides cellulosolvens* (*B. cellulosolvens*), enzymes are associated with type II dockerins, while scaffoldins are associated with type I dockerins (Bayer et al., 2008; Cameron et al., 2015; Duarte et al., 2021; Noach et al., 2005). Type III pairs are specific to ruminococcal cellulosomes and differ from the type I and II found in *Clostridium* species (Duarte et al., 2023; Voronov-Goldman et al., 2015). Understanding these modular and specific cohesin-dockerin interactions lays the foundation for harnessing and redesigning cellulosomes as modular synthetic scaffolds, opening avenues for customizable enzyme complexes in synthetic biology.

### 3.1 Cohesin-dockerin binding modes and their structural determinants

The binding mode of a given dockerin is dictated by its structural features and the conservation or divergence of key interacting residues. Type I dockerins are well-documented for their symmetrical binding surface, which supports dual binding modes. They contain two EF-hand-like Ca^2+^-binding motifs, each with symmetrical antiparallel *α*-helices flanking the conserved Ca^2+^-binding residues (aspartate and asparagine) and coordination patterns (Adams et al., 2005; Pagès et al., 1997; Pinheiro et al., 2009). This duplication creates a symmetrical cohesin-binding surface with residues critical for species-specific recognition, enabling dual binding in two orientations (180° apart). Dual binding mode increases flexibility and efficiency in enzyme assembly within the cellulosome and thus provides enhanced function, avoidance of steric clashes, and adaptation to substrates (Carvalho et al., 2007; Carvalho et al., 2003; Chen et al., 2014; Pagès et al., 1997; Pinheiro et al., 2008). In this mode, the cohesin structure remains mostly unchanged, while the dockerin’s loop–helix–helix–loop–helix motif undergoes conformational changes, yet retaining its EF-hand coordination with Ca^2+^ ions. Dual-binding mode was recently proposed to be allosterically regulated. Two alternative binding conformations in type-I cohesin-dockerin pairs were identified, and their binding mode mediated by the isomerization state of a single proline residue. While the exact determinants of binding mode equilibrium remain unclear, they likely depend on the specific cohesin-dockerin pair (Vera et al., 2021).

Until recently, type II cohesin-dockerin pairs (typically involved in cell wall attachment) were considered to adopt a single-binding mode. In single binding mode, only one of the dockerin interfaces supports the formation of the cohesin-dockerin complex, as seen for type II *C. thermocellum* dockerin (Adams et al., 2006) or adaptor scaffoldins in *R. flavefaciens* (Bule et al., 2016). However, recent work revealed a dual-binding mode of type II dockerin from *B. cellulosolvens* (Duarte et al., 2021). This finding challenges the previous belief that a dockerin’s binding mode depends on its type or function. In fact, both type I and type II complexes, whether involved in enzyme recruitment or cell-surface anchoring and scaffoldin assembly, exhibit dual-binding modes. This was further confirmed in *Ruminococcus*-specific type III complexes (Duarte et al., 2023). The structure of type III dockerins exhibits less symmetry compared to type I and II dockerins found in *Clostridium* species and lacks the second canonical Ca^2+^-binding loop (Bule et al., 2018b; Duarte et al., 2023; Rincon et al., 2007; Rincon et al., 2005; Rincon et al., 2003; Salama-Alber et al., 2013; Voronov-Goldman et al., 2015). Nevertheless, it demonstrates similar behavior, maintaining responsiveness to Ca^2+^ and a strong affinity for the cohesin on the scaffoldin (Karpol et al., 2013). Type III-specific recognition “hot spots” were identified by switching the specificity of ScaA cohesin to mimic type I cohesin interaction (Weinstein et al., 2015). Notably, Duarte et al. (2023) described the crystal structure of the adaptor scaffoldin ScaH-borne dockerin bound to the cohesin of the anchoring scaffoldin ScaE, demonstrating an unexpected dual-binding mode of a type III complex (Duarte et al., 2023).

All in all, the dual-binding mode is much more widely distributed than initially proposed (Bule et al., 2018a). There is no definitive evidence for selective pressure favoring the dual-binding mode. Species like *R. flavefaciens* can assemble functional cellulosomes using only single-binding modes, suggesting the dual-binding mechanism may lack universal evolutionary relevance (Brás et al., 2012; Vera et al., 2021; Yao et al., 2020). Overall, the active regulation of dockerin-binding orientation and the complexity of systems like *Bacteroides cellulosolvens* suggest that single vs. dual-binding modes in cohesin-dockerin complexes are independent of type or function (enzyme recruitment vs. cell attachment/scaffoldin assembly; Duarte et al., 2021). Instead, these modes may be linked to cellulosome size and complexity, with larger systems requiring greater flexibility for assembly and substrate access. The effect of cellulosome size on catalytic efficiency was investigated recently using recombinantly assembled cellulosomes. Primary and secondary scaffoldins containing 1, 3, or 5 type I/II cohesin domains were synthesized and assembled with 1, 3, 5, 9, 15, or 25 cellulase molecules from GH families 5, 9, and 48. These complexes were attached to the cellulose by CBM3a. It was found that increasing cellulosome complexity enhances hydrolysis efficiency. Cellulosomes with 9, 15, or 25 cellulases showed similar activity, suggesting synergy saturated at 9 subunits in this system (Chen and Ge, 2018). However, in cellulolytic bacteria like *C. thermocellum*, which produce dozens of diverse cellulases, synergy continues to increase with higher enzyme diversity—up to 40 enzymes have shown enhanced effects (Hirano et al., 2016), highlighting the need for further investigation.

### 3.2 Multi-dockerin modules and cellulosome complexity

Generally, cellulosomal scaffoldins are composed of multiple tandem cohesin modules, each capable of binding different enzymes, most of which have only a single dockerin (Carvalho et al., 2007). Nevertheless, rare double- and multiple-dockerin modules have been identified in some cellulosome-producing bacteria. For example, in *C. thermocellum*, double-dockerins were identified, either with no partner or bearing a protease domain (Chen et al., 2024; Chen et al., 2019). Structural analysis of the S8 protease-associated double-dockerin module dDoc_0689 from *C. thermocellum* revealed that its two dockerins form a stable interface through hydrophobic interactions and hydrogen bonds with limited mobility. The first dockerin module displays a unique intramolecular clasp and exhibits binding preference to the cohesin of the cell-bound scaffoldin ScaD, while its second dockerin module remains functionally ambiguous. These findings suggest that dDoc_0689 may facilitate protease anchoring to the bacterial cell surface rather than integrating into primary enzyme-scaffoldin complexes (Chen et al., 2024). These double-dockerin proteins are conserved across different *C. thermocellum* strains and their expression has been confirmed by transcriptomic and proteomic studies (Raman et al., 2011; Wilson et al., 2013). Furthermore, genes encoding triple, sextuple and septuple dockerins have been identified in the genomes of some other bacterial species, for example *R. flavefaciens* and *Oscillospiraceae bacterium* J5864_00540 (Chen et al., 2024; Dassa et al., 2014) and also fungi (Gilmore et al., 2020). These multi-dockerin proteins might provide even more complexity for the assembly of cellulosomes, yet their structure and function are still unknown. Unraveling the conformation of these regions will not only deepen our understanding of cellulosomes but also contribute novel elements to synthetic biology and biotechnology (Chen et al., 2024).

### 3.3 Specificity and cross-species interactions of cohesin-dockerin complexes

The cohesin-dockerin interaction has generally been considered species-specific. The specificity of type I and type II cohesin-dockerin interactions is believed to prevent cross-reactivity, ensuring precise cellulosome assembly and cell surface attachment. This selectivity is governed by subtle differences in binding site topology and the lack of sequence identity in *β*-strand sequences that comprise the core of the dockerin binding site (Alber et al., 2009; Cameron et al., 2015; Carvalho et al., 2005; Carvalho et al., 2003; Noach et al., 2005), with a few key residues governing cohesin-dockerin specificity (Nakar et al., 2004). In type I cohesin-dockerins from *C. cellulolyticum* and *C. thermocellum*, species-specific recognition has been attributed to apolar interactions between the key residues centered around a hydrophobic pocket on the surface of the cohesin. In *C. cellulolyticum*, this pocket is formed by Leu87 and Leu89, which is occupied, in the two binding modes, by the dockerin residues Phe19 and Leu50, respectively (Pinheiro et al., 2008). Using a combination of computational and experimental methods, two types of hot spot residues were identified in *C. thermocellum* cohesin: affinity hot spots like Leu83, which enhance binding strength, and specificity hot spots like Asn37, which determine partner selectivity (Slutzki et al., 2015). When Asn37 mutants were investigated, it was found that N37A binds both *C. thermocellum* and *C. cellulolyticum* dockerins promiscuously, while N37L shifts specificity toward *C. cellulolyticum*, reducing binding to the native partner (Slutzki et al., 2015). Nevertheless, in contrast to the type I, type II cohesin-dockerin pairs have a relatively extensive cross-species plasticity, which is common among the simple cellulosome systems of mesophilic clostridia (Haimovitz et al., 2008). Recently, a higher affinity for a cohesin from *C. cellulolyticum* than for those in *C. thermocellum* was detected in a study where a rare double-dockerin module from *C. thermocellum* was tested (Chen et al., 2024). An exception in species specificity was also observed in *C. thermocellum* dockerin interaction with *C. josui* cohesin (Jindou et al., 2004). However, biological relevance of this lack of species specificity is still unclear. Inter-species cohesin-dockerin interactions support the pan-cellulosome concept (Dassa et al., 2017), allowing microbial communities in shared ecosystems to assemble “symbiotic cellulosomes.” This proposed cooperative strategy enhances enzymatic diversity, particularly under extreme conditions, without additional resource investment (Chen et al., 2024).

### 3.4 Biotechnological applications and engineering potential of cohesin-dockerin systems

As already detailed in the previous sections, the structural flexibility (3.1), multi-dockerin complexity (3.2), and specificity patterns (3.3) of cohesin-dockerin interactions offer powerful tools for synthetic biology. These features enable precise design of modular, multi-enzyme assemblies with controllable binding and functionality, paving the way for advanced biotechnological applications and engineered biomaterials.

Recent genomic analysis of 305,693 sequenced bacterial genomes identified 33 bacterial species with the genomic potential to produce cellulosomes (Minor et al., 2024). A high number of cohesins and dockerins identified are not only valuable as building blocks for DCs but also hold promise for the broader design of protein scaffolds enabling multi-enzyme assembly (reviewed in Gad and Ayakar, 2021). By combining cohesin-dockerin pairs from different species, it is possible to precisely control the enzyme composition, spatial arrangement, and stoichiometry within the DC (Vanderstraeten et al., 2022a). Advancements in protein engineering supported by computational modeling have led to the development of cohesin-dockerin pairs with tailored specificity, improved binding affinities, and increased stability under environmental conditions (Anandharaj et al., 2020; Bomble et al., 2011; Borne et al., 2020; Dorival et al., 2022; Duarte et al., 2023; Gilmore et al., 2020; Vazana et al., 2013; Vera et al., 2021; Wojciechowski et al., 2018). One notable achievement in this area is the construction of the largest known cellulosome complex, displaying 63 enzymes on the surface of *Kluyveromyces marxianus*. This system supports the modular addition of enzymes, creating a cellulolytic host for ethanol production. In this study, scaffoldins containing three, six, or nine cohesin modules were tested. Experiments revealed that enzyme synergism—and thus sugar release from highly crystalline substrates such as Avicel—was significantly influenced by cohesin number. In contrast, the impact was less pronounced when phosphoric acid-swollen cellulose (PASC) was used (Anandharaj et al., 2020).

Further insights into cellulosome dynamics were provided by Borne et al. (2020), who demonstrated that a fully assembled cellulosome can be functionally reprogrammed. They showed that the stability of preassembled enzyme–scaffoldin complex is governed by a key dockerin aminoacid residue at position 22. This position determines whether the interaction with the cohesin is reversible or nearly irreversible. Site-directed mutagenesis of this residue enables the conversion between these two binding states (Borne et al., 2020). Leucine residues at positions 22 and 58 are highly conserved among dockerin-bearing proteins across multiple bacterial species (Dassa et al., 2017). They are located at the termini of the *α*-helices in both dockerin segments (Pinheiro et al., 2008). Due to the dual-binding mode of cohesin–dockerin interactions, one of these residues is consistently positioned at the cohesin interface (Borne et al., 2020). Their conservation suggests a general mechanism underlying cellulosome dynamics across bacterial species and highlights the biotechnological potential of replacing specific enzymatic subunits through competition between dockerin variants (Borne et al., 2020).

Further, pH-dependent dual-binding-site switch in *C. acetobutylicum* cohesin-dockerin pair was recently discovered. This marks the first report of a pH-regulated protein–protein interaction switch and represents a model of biological regulation and a novel strategy for designing pH-responsive protein devices and biomaterials for biotechnology (Yao et al., 2020). In addition, dockerins’s electrostatic profile alteration can enhance module affinity, providing a versatile platform for designing high-affinity technologies in industrial and research applications (Duarte et al., 2023). The cohesin-dockerin interaction is among the strongest non-covalent interactions in nature (Duarte et al., 2021). Its mechanical stability also influences cellulosomes enzymatic activity (Stahl et al., 2012). Mechanical forces have been shown to influence cohesins located on the scaffoldin (in the connecting region between the cell and cellulose anchoring points). This might lead to forced unfolding of cohesins and therefore to dockerin (and enzyme) release, resulting in a negative effect on cellulosome activity (Galera-Prat et al., 2020; Galera-Prat et al., 2018). In a study by Galera-Prat et al. (2020), the cellulolytic activity of bound cellulosomes was compared to that of a free complex after the exposure to mechanical stress using magnetic stirring under high agitation conditions. Specifically, monovalent cellulosomes were designed bearing four different single cohesins with known mechanical stability. They were attached to polystyrene microparticles to mimic cell surface attachment. It was found that a cohesin with a low mechanical stability in a scaffoldin’s connecting region decreases the cellulosome’s activity compared to one with the higher stability. This highlights mechanical stability as an emerging industrial parameter in biotechnology. Specifically, in two-point attachment systems, cohesins between anchoring points should have high mechanical stability to preserve activity of the cellulosome. Notably, these principles may also explain the evolution of natural cellulosome architectures (Galera-Prat et al., 2020).

## 4 Cell-surface anchorage

For the majority of cellulosomes described so far, attachment to the bacterial cell surface relies on calcium-mediated interactions between type II dockerin modules of the cellulosomal scaffoldin and cohesin modules of cell-surface proteins anchored in the peptidoglycan layer. Structural and biochemical studies of clostridial cell surface dockerin-cohesin complex revealed two cohesin-binding interfaces with differential specificities (Adams et al., 2006). Type II dockerins of primary scaffoldins from *C. thermocellum* and *C. cellulolyticum* ScaA and CipB feature two distinct cohesin-binding surfaces that recognize different cohesin partners, while in *A. cellulolyticus*, the dual-binding mode of the type II dockerin module provides enhanced flexibility in cohesin recognition and allows greater versatility for efficient cellulosome assembly (Brás et al., 2016).

### 4.1 Anchoring scaffoldins: sortase- and SLH module-based mechanisms and beyond

Cell surface-anchoring scaffoldins are typically embedded in the peptidoglycan either through sortase-mediated covalent attachment or by noncovalent binding of scaffoldins via S-layer homology (SLH) modules. In the sortase-mediated mechanism, as described for the scaffoldin ScaE from *R. flavefaciens*, a sortase recognition motif (LPXTG) is cleaved by the sortase at the cell surface and the protein is covalently linked to the cell wall (Rincon et al., 2005). This well-characterized process is common in gram-positive bacteria (reviewed in Bhat et al., 2021). The second mechanism involves non-covalent interactions between polysaccharides protruding from the peptidoglycan and SLH modules on the scaffoldins. Herein, cohesin domains are connected to SLH modules through flexible linkers (Zhao et al., 2006). In *C. thermocellum,* four novel anchoring scaffoldins ScaB, ScaC, ScaD and ScaF were identified (Brás et al., 2016). Interestingly, ScaF is the only one that contains an additional module of unknown function (ScaF-X) between the cohesin and SLH modules, with no homologs found in the Protein Data Bank (PDB) database. NMR-based studies suggest that ScaF-X is a well-folded and potentially functional protein module, holding promise as a new element in synthetic biology and biotechnology application, although further research is needed to elucidate its role (Li et al., 2021).

Complex cellulosome producers like *Acetivibrio cellulolyticus* (*A. cellulolyticus*) harbor numerous genes encoding CBM3-SLH fusion proteins (Figure 2), which were proposed to facilitate microbial attachment to the cellulose (Minor et al., 2024; Wang et al., 2022). In addition to sortase recognition and SLH motifs, other domains may play a role in cellulosome attachment to the cell wall. In *B. cellulosolvens* genome, several novel domains within its scaffoldins like PA14 domain in ScaU, cadherin in ScaD, VCBS in ScaH3, and PPC domains in ScaO and ScaP, were recently identified (Zhivin et al., 2017). These domains are typically associated with functions like carbohydrate binding, cell adhesion, and protein–protein interactions (Finn et al., 2017). Their presence suggests potential mechanisms for cell wall anchoring and substrate recognition beyond the classical SLH-based system.

Unlike the more complex cellulosomes, simple scaffoldins such as those found in *R. cellulolyticum* typically lack classical cell wall-binding modules like SLH domains. While they do contain cohesin and dockerin domains necessary for enzyme assembly, their mode of anchoring to the cell surface has remained unclear. Recent findings suggest that the N-terminal CBM3a domain of the primary scaffoldin CipC can bind to the bacterial surface and may contribute to cellulosome localization (Tao et al., 2022). Additionally, while X2 domains do not directly bind to the cell wall or cellulose in *R. cellulolyticum*, they are thought to influence the spatial organization of the cellulosome. As such, they may modulate interactions with cellulosic substrates or other components of the cell surface (Kosugi et al., 2004; Tao et al., 2022). In other species such as *C. cellulovorans*, X2 domains have been implicated in scaffoldin surface display, especially when present alongside SLH modules (Tarraran et al., 2021). These findings indicate that CBM3a and X2 domains may contribute to alternative modes of surface localization in simple cellulosomes, though further research is needed to clarify their precise roles and potential cooperative functions.

### 4.2 Cell-free scaffoldins

Most cellulosomes were found to be attached to cell surfaces, but cell-free scaffoldins have also been observed in species like *C. thermocellum*, *Clostridium clariflavum*, and *A. cellulolyticus* (Artzi et al., 2015; Raman et al., 2009; Xu et al., 2016; Zhivin-Nissan et al., 2019). Some secrete cell-free cellulosomes with multi-cohesin scaffoldins and dockerin-tagged GH enzymes to degrade lignocellulosic biomass. Certain species, including *R. sufflavum*, *Clostridium* sp. HBUAS56017 and *Clostridium bornimense*, have been reported to produce scaffoldins without dockerin or cell wall-binding modules, suggesting secretion into the environment (Minor et al., 2024). Cell-free scaffoldins were suggested to play an important role in degradation of remote cellulosic substrates (Zhivin-Nissan et al., 2019). While the potential for free cellulosomes to facilitate cooperative lignocellulose breakdown is intriguing, the regulatory mechanisms governing their release remain unclear. Further research is needed to determine the mechanism(s) that control the secretion of free cellulosomes, and how this regulation might optimize their deployment for synergistic degradation.

### 4.3 Biotechnological applications of cell surface display systems

Surface-display technologies enable the functional assembly of DCs on non-cellulolytic microbial hosts, generating whole-cell biocatalysts capable of lignocellulosic biomass degradation and consolidated bioprocessing. This strategy integrates enzymatic hydrolysis with downstream metabolic conversion in a single microbial chassis. In food and health-related applications, lactic acid bacteria such as *Lactococcus lactis* and *Lactobacillus plantarum* offer the advantages of GRAS (Generally Recognized As Safe) status and probiotic potential. For instance, scaffoldins from *C. cellulovorans* containing different combinations of X2 and SLH domains were successfully displayed on the surface of *Lactococcus lactis* (Tarraran et al., 2021). This suggests a shared binding mechanism mediated by components of the thick peptidoglycan layer (Tarraran et al., 2021), which is known to be interwoven with proteins and glycopolymers such as teichoic and lipoteichoic acids (Desvaux et al., 2006). Similarly, DCs have been assembled on the surface of *Lactobacillus plantarum* (Stern et al., 2018). *Bacillus subtilis*, another GRAS-designated and industrially robust Gram-positive host, was used to anchor a trifunctional minicellulosome via a cell wall-binding domain derived from its endogenous hydrolase LytE (You et al., 2012). Yeasts, particularly suited for biofuel production, have also been extensively explored (reviewed in Lamote et al., 2023; Li et al., 2024). For example, in *Saccharomyces cerevisiae*, a tetravalent DC displayed via adaptor scaffoldins led to a several-fold enhancement in cellulose hydrolysis and ethanol yield relative to free enzymes (Tsai et al., 2013). In a study by Tang et al. (2018), a yeast surface display system was developed in which cellulosomal components were anchored via disulfide bonds to a yeast cell wall protein, significantly improving display efficiency in *S. cerevisiae* (Tang et al., 2018). Recently, *Pichia pastoris* has been engineered as a whole-cell biocatalyst for carboxymethyl cellulose to ethanol conversion. Herein, an ultra-high-affinity IM7/CL7 system from *E. coli* was used to display minicellulosomes assembled *in vitro* from endoglucanase, exoglucanase, β-glucosidase and CBM. To date, this is the first example of engineered yeast enabling efficient and direct conversion of carboxymethyl cellulose to ethanol with titers reaching 5.1 g/L (Dong et al., 2020). Further, probiotic yeast *Kluyveromyces marxianus* was engineered to produce and display the largest cellulosome complex that can accommodate up to 63 enzymes. Herein, the surface attachment of the designed scaffoldin was achieved by replacing its SLH domain with the glycosylphosphatidylinositol (*Sc*GPI), a cell-surface protein from *S. cerevisiae* (Anandharaj et al., 2020). Finally, Gram-negative bacteria have also been adapted for surface display, despite structural limitations posed by their outer membrane. For example, a cohesin-dockerin pair from *Archaeoglobus fulgidus* was employed to attach a dimeric *α*-neoagarobiose hydrolase to the surface of *E. coli* cells (Ko et al., 2021). Altogether, these platforms extend cellulosome display across diverse microbial hosts and hold strong promise for industrial biotechnology, sustainable bioenergy production, and the development of next-generation probiotics.

## 5 Catalytic subunits and the functional interplay of cellulosomal components

One of the key advantages of cellulosomes over free enzymes lies in the close spatial proximity of their enzymatic components. This proximity enables coordinated and efficient degradation of cellulose and other plant cell wall polysaccharides. It also facilitates substrate channeling, minimizes product inhibition and enhances overall efficiency. Compared to free enzymes, cellulosomes demonstrate superior substrate conversion due to the synergistic action of their enzyme assemblies (Artzi et al., 2017; Vazana et al., 2010).

### 5.1 Catalytic repertoire of cellulosomes

Cellulosomes typically incorporate a wide array of carbohydrate-active enzymes (CAZymes) that contribute to the degradation of plant cell walls (Table 1). These include: (1) GHs—notably from families such as GH5, GH9, GH10, GH11, GH26, and GH48—which hydrolyze glycosidic bonds in cellulose, hemicellulose, and other glycans (e.g., xylanases, mannanases, xyloglucanases, glucanases), (2) carbohydrate esterases (CEs)—which remove ester-linked substituents (e.g., acetyl or feruloyl groups) that can hinder GH access to their substrates, (3) polysaccharide lyases, that cleave polysaccharides through non-hydrolytic mechanisms, such as *β*-elimination, facilitating the degradation of pectins and other acidic polysaccharides, and in some cases, (4) non-CAZy accessory proteins, such as proteases, protease inhibitors, and expansin-like proteins, which may modulate enzyme accessibility or cell wall structure (Artzi et al., 2017; Gharechahi et al., 2023; Lombard et al., 2025). Altogether, this complex and modular enzymatic architecture underlies the remarkable versatility and efficiency of the cellulosomal system.

**Table 1:** Catalytic subunits identified as parts of native bacterial cellulosomes.

Substrate	Enzyme function	EC number	Family examples	References
Cellulose	Endoglucanase (endo-1,4-β-glucanase)	EC 3.2.1.4	GH5, GH8, GH9, GH24, GH44	Aburaya et al. (2019); Blouzard et al. (2010); Esaka et al. (2015); Gold and Martin (2007); Hirano et al. (2016); Matsui et al. (2013); Morisaka et al. (2012); Raman et al. (2009); Vodovnik et al. (2013); Zhivin-Nissan et al. (2019)
	Exoglucanase (cellobiohydrolase)	EC 3.2.1.91	GH9, GH48	
Hemicellulose	Endo-β-1,4-xylanase	EC 3.2.1.8	GH5, GH10, GH11, GH30	Aburaya et al. (2019); Blouzard et al. (2010); Esaka et al. (2015); Gold and Martin (2007); Han et al. (2004); Hirano et al. (2016); Matsui et al. (2013); Morisaka et al. (2012); Murashima et al. (2002); Raman et al. (2009); Vodovnik et al. (2013); Zhivin-Nissan et al. (2019)
	Mannanase	EC 3.2.1.78	GH5, GH26	
	β-xylosidase	EC 3.2.1.37	GH3, GH43	
	α-arabinofuranosidase	EC 3.2.1.55	GH43, GH51, GH62	
	α-glucuronidase	EC 3.2.1.139	GH67, GH115	
	Acetyl xylan esterase	EC 3.1.1.72	CE1, CE3, CE4, CE6, CE12	
	Feruloyl esterase	EC 3.1.1.73	CE1, CE6, CE7	
	Xyloglucanase	EC 3.2.1.151	GH74	
Pectin	Rhamnogalacturonan hydrolase	EC 3.2.1.171	GH28	Aburaya et al. (2019); Blouzard et al. (2010); Gold and Martin (2007); Hirano et al. (2016); Morisaka et al. (2012); Pagès et al. (2003); Raman et al. (2009)
	Polygalacturonase	EC 3.2.1.15	GH28	
	Endo-1,4-β-galactanase	EC 3.2.1.89	GH53	
	Rhamnogalacturonan lyase	EC 4.2.2.23	PL4, PL11	
	Pectate lyase	EC 4.2.2.2	PL1, PL9	
	Rhamnogalacturonan acetyl esterase	EC 3.1.1.86	CE12	
β-Glucans	Lichenase (β-1,3-1,4-glucanase)	EC 3.2.1.73	GH16	Gold and Martin (2007); Hirano et al. (2016)
Proteins /peptides	Protease/peptidase	EC 3.4.21.-EC 3.4.22.-	Peptidase S8 (subtilisin-like) Cysteine peptidase	Levy-Assaraf et al. (2013); Morisaka et al. (2012)
Chitin	Chitinase	EC 3.2.1.14	GH18	Hirano et al. (2016); Zverlov et al. (2002)

Efficient hydrolysis of β-glycosidic bonds in cellulose relies on the coordinated, synergistic action of multimodular enzymes with two types of catalytic domains: endoglucanases and exoglucanases. Endoglucanases cleave internal β-1,4-bonds within the cellulose polymer generating shorter oligosaccharides. Exoglucanases cleave the chains progressively from either the reducing or non-reducing end, releasing cellobiose or cellotetraose units. β-glucosidases then hydrolyze cellobiose into glucose, alleviating product inhibition and making glucose available for microbial metabolism (reviewed in Datta, 2024; Ranjan et al., 2023; Sidar et al., 2020). This synergy is quantitatively reflected in a degree of synergistic effect often exceeding the sum of individual enzyme activities, with optimal enzyme ratios, substrate properties, and intermolecular proximity further enhancing hydrolysis efficiency.

The complexity of hemicellulose requires a diverse set of hemicellulases. Endoxylanase and xylosidases degrade the xylan backbone, while accessory enzymes such as α-L-arabinofuranosidases remove arabinose side chains from arabinoxylans. Acetyl xylan esterases remove acetyl groups from xylan chains, and ferulic acid esterases cleave ester linkages between hemicellulose and lignin. Additionally, α-glucuronidases remove glucuronic acid side chains from glucuronoxylans, and mannanases and β-mannosidases facilitate the degradation of mannan rich hemicelluloses.

Alongside (hemi)cellulose and pectins, lignin represents a key structural component of plant cell walls. Unlike cellulolytic enzymes, lignin-degrading oxidative enzymes, such as laccases, lignin peroxidases and manganese peroxidases are exclusively produced by aerobic organisms like white rot fungi and certain bacteria and have therefore not been identified within native bacterial cellulosomes (reviewed in Artzi et al., 2017; Gharechahi et al., 2023; Wang et al., 2019). Nevertheless, advances in synthetic biology have enabled the engineering of “designer cellulosomes” that incorporate lignin modifying enzymes (Davidi et al., 2016).

A diverse array of proteins beyond traditional carbohydrate-active enzymes has been identified within cellulosomes, contributing to enzymatic synergy and functional regulation. Notably, expansin-like proteins have been discovered in the cellulosome system of *Clostridium clariflavum*. Two such proteins, CclEXL1 (Clocl_1862) and CclEXL2 (Clocl_1298), contain type I dockerin modules, suggesting their incorporation into the cellulosome complex. Proteomic analyses have confirmed the expression of CclEXL1 under specific growth conditions, with its dockerin module exhibiting selective binding to type I cohesins, particularly the cohesin of scaffoldin ScaG (Artzi et al., 2016). Functionally, CclEXL1 binds preferentially to microcrystalline cellulose and demonstrates a pronounced loosening effect on cellulose fibers. This enhances the enzymatic hydrolysis of cellulose by both native cellulosomes and individual cellulases such as GH48 and GH9 (Artzi et al., 2016). The biological role of bacterial expansins remains under investigation (de Sandozequi et al., 2022), but their incorporation into cellulosomes suggests they play a supportive role in optimizing substrate accessibility (Cosgrove, 2017). Furthermore, both free and dockerin-containing expansin-like proteins from *Bacillus subtilis* and *C. clariflavum* have been shown to promote cellulose degradation by native cellulosomes. This synergistic effect is amplified when these proteins are integrated into trivalent DCs, further enhancing their cellulolytic performance (Artzi et al., 2016; Chen C, et al., 2016).

Further on, a protease domain in double dockerin-containing proteins was identified in various cellulosome-producing bacterial species (Chen et al., 2024). It was proposed that it may facilitate the maturation, activation, and turnover of cellulosomal components, ensuring optimal enzyme function. In *R. flavefaciens*, putative cysteine peptidase that binds to the surface-anchoring ScaE cohesin via its X-dockerin modular dyad was identified (Levy-Assaraf et al., 2013). In addition, the structure of putative S8 protease with a tandem bimodular double-dockerin from *C. thermocellum* was determined. These dockerins were able to bind to various scaffoldins from different species, implying that rather than being directly involved in biomass degradation, cellulosomal peptidases may play a role in cellulosomal posttranslational processing or cellular nitrogen recycling from secreted proteins (Chen et al., 2024).

Interestingly, cellulosome-localized protease inhibitors were also identified in *Clostridia*. In *C. thermocellum*, serpins were suggested to play a protective role in cellulosomes by inhibiting subtilisin-like proteases, safeguarding the integrity of the complex (Kang et al., 2006; Ó Cuív et al., 2013). Similarly, in cellulosomes of *C. cellulolyticum*, a dockerin-containing protease inhibitor Dpi was discovered that protects key cellulosomal cellulases from proteolysis (Xu et al., 2014). On the other hand, proteolytic mechanisms may allow cellulosome-producing bacteria to recruit specialized enzymes from other organisms in the same ecosystem. This saves energy and resources and could also regulate cellulosome assembly in different species (Chen et al., 2024; Haimovitz et al., 2008). However, the exact roles of these proteases and their specific locations within the cellulosome need further experimental validation.

### 5.2 Spatial and structural principles guiding designer cellulosome construction

A deep understanding of cellulosomal architecture is essential for designing efficient, tailored DCs. The diverse enzyme composition in cellulosomes enables effective plant polysaccharide degradation, with the cellulosomal architecture minimizing the diffusion and enhancing the uptake of the degradation products (Arora et al., 2015; Vodovnik and Marinšek-Logar, 2010). For enhanced synergistic activity, the position of the enzymes in the scaffoldin, enzymes functionality and flexibility of scaffoldin and enzyme-dockerin linkers are of significant importance. Optimal distances between the active sites of different enzymes on the protein scaffold enable effective substrate channeling, promoting sequential reactions over diffusion (Deng et al., 2020). In a study by Stern et al. (2015), the optimal position of recombinant processive endoglucanase Cel9A from *T*. *fusca* was determined relative to two additional enzymes: the *C. thermocellum* exoglucanase Cel48A and the *C. cellulolyticum* endoglucanase Cel5A. The optimal order was determined as follows: processive endoglucanase Cel9A (GH9), exoglucanase Cel48A (GH48) and endoglucanase Cel5A (GH5). A GH9 endoglucanase proximity to the GH5 endoglucanase negatively affected overall degradation efficiency. This is in accordance with the fact that these two enzymes possess similar functionalities. Thus, their immediate proximity may cause unproductive competition between the two catalytic sites of the two functionally similar enzymes (Stern et al., 2015). This is in line with the results of a similar study on *C*. *cellulolyticum*, where a successful enzyme combination was obtained using a chimeric scaffoldin in which the GH9 processive endoglucanase was placed on the scaffoldin together with the GH48 exoglucanase and away from the GH5 endoglucanase (i.e., GH5-GH48-GH9; Fierobe et al., 2005). To determine if docking enzyme size affects DC activity, Vanderstraeten et al. (2022a) used a designed cellulosome complex with three enzymes: a smaller mannanase (47 kDa), a galactosidase (54 kDa), and a larger mannosidase (104 kDa). Placing the larger mannosidase between the smaller enzymes did not reduce mannose monomer production (Vanderstraeten et al., 2022a). Similarly, Vazana et al. (2013) found no preferred modular arrangement when incorporating three *C. thermocellum* enzymes of varying sizes—an exoglucanase Cel48S (82 kDa), an endoglucanase Cel8A (52 kDa), and an endoglucanase Cel9K (99 kDa)—into a DC (Vazana et al., 2013). These results suggest that docking enzyme size is not a primary factor influencing substrate degradation efficiency of the complex. The position of the CBM of the scaffoldin also proved to be an important factor in cellulosome functionality. In case of the cellulosomes incorporating recombinant enzymes originating from *T. fusca*, it was shown to be optimal when placed close to the processive endoglucanase and at the extremity of the scaffoldin (either at the N- or at the C- terminus), allowing more freedom in movement (Hammel et al., 2004; Stern et al., 2015).

In order to adapt to the complexity of the substrate, cellulosome requires maximal flexibility, which is mainly provided by the intermodular linkers of scaffoldin subunits. Structural analyses, including crystal structures and SAXS studies (Currie et al., 2012; Hammel et al., 2004; Molinier et al., 2011; Noach et al., 2009), have revealed that these linkers confer the necessary conformational adaptability to scaffoldins, facilitating efficient substrate degradation. Vazana et al. (2013) investigated the impact of intermodular linker length on DC activity. Scaffoldins with no linker, short linkers (5 aminoacids) and long native linkers (27–35 amino acids) were compared, and DCs with the long linker were found to achieve higher levels of activity on both pure and complex substrates. This enhancement was attributed to increased flexibility and optimal spatial arrangement of catalytic modules (Vazana et al., 2013). In addition, ScaH, a *R. flavefaciens* adaptor scaffoldin, can work as a variable length spacer to avoid clashes between cellulosomal units on the bacterial surface, thereby promoting efficient assembly of complex cellulosomal structures (Duarte et al., 2023). In modular GHs, the length and flexibility of the linker between the CBM and the catalytic modules were found to be critical for the catalysis. Studies on GH5 family cellulase 5A from *B. subtilis* (Ruiz et al., 2016) and GH45 family endoglucanase from *Rhizopus stolonifera* (Tang et al., 2015) have demonstrated that deviations from optimal linker properties can impair enzyme function, underscoring the importance of precise inter-domain spacing and flexibility. However, the influence of linker properties can be enzyme-specific. Caspi et al. (2009) converted the modular *T. fusca* cellulase Cel5A to a cellulosomal configuration by replacing its CBM with a dockerin module from either *C. thermocellum* or *R. flavefaciens*. They found that varying the linker length between the catalytic domain and the dockerin had minimal impact on enzymatic activity (Caspi et al., 2009). This suggests that some enzymes are less sensitive to linker variations. Kahn et al. (2019) explored the effects of introducing long linkers between catalytic domains and dockerin modules in *Caldicellulosiruptor bescii* enzymes. They found that a longer linker enhanced GH9 activity but had no effect on GH48-containing complexes. The differential impact was attributed to the absence of glycosylation in recombinant linkers, which, unlike in native enzymes (Chung et al., 2015), may affect flexibility and protection from proteolysis, influencing enzyme stability and function (Kahn et al., 2019).

Recently, a trivalent DCs carrying mannanase, mannosidase and galactosidase was constructed and optimized for galactomannan degradation. By testing linkers that differ in flexibility and length on the three enzymes, it was found that direct enzyme-dockerin fusion with no linker significantly decreased the enzyme activity. In addition, the optimal linker type and length was found to be enzyme-dependent (Vanderstraeten et al., 2022b). Furthermore, a lytic polysaccharide monooxygenase (LPMO) catalytic domain combined with a linker region and CBM domain was used as a model to investigate the effect of the linker on enzyme activity. Herein, the linker region was found to be important for protein thermostability, binding of and activity toward recalcitrant polysaccharide substrate (Srivastava et al., 2022).

Altogether, these results highlight the importance of architectural parameters like linker flexibility and length, positioning of the catalytic units within the scaffoldin, and enzymes functionality in enhancing the performance of cellulases and/or cellulosomes. Understanding how these factors influence cellulosomal function provides a crucial foundation for the rational design of DCs. By strategically engineering these features, it is possible to optimize enzyme synergy, improve substrate accessibility, and increase catalytic efficiency. Insights gained from natural systems and synthetic assemblies enable the construction of tailored DCs suited for specific substrates or industrial applications.

## 6 Advances in designer cellulosome construction

**Figure 3:**
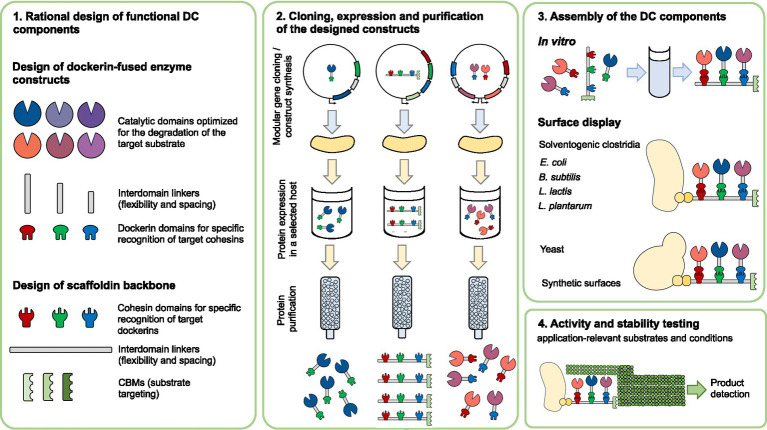
Overview of designer cellulosome construction and assembly. A modular workflow for the design, production, and assembly of a (synthetic) designer cellulosome. Scaffoldins with species-specific cohesins and CBMs are combined with dockerin-tagged catalytic domains to form multifunctional complexes. Following expression and purification, components are assembled either *in vitro* or via surface display in industrially-relevant strains. The performance of the resulting complexes is than assessed under application-relevant conditions. CBM: Carbohydrate-binding module, DC- designer cellulosome.

Enzymatic efficiency in the degradation of complex biomass can be significantly enhanced by incorporating heterologous enzymes or cellulosomal components into DC assemblies. These engineered complexes enable precise control over enzyme composition, spatial organization, and overall stability, thereby achieving catalytic performances that surpass those of native systems. In addition to the strategies discussed above, and summarized in Figure 3, several notable approaches have been developed to further enhance the enzymatic efficiency of DCs. One effective strategy involves increasing the enzyme load within a single cellulosomal complex. For instance, in *Acetivibrio cellulolyticus*, the adaptor scaffoldin ScaB mediates the incorporation of up to four ScaA scaffoldins into a single cellulosome, substantially expanding the enzymatic repertoire contained within one assembly (Brás et al., 2016). Another innovative approach was demonstrated by Gilmore et al. (2020), who engineered chimeric enzymes by fusing fungal dockerin domains with GH catalytic modules from the hyperthermophilic bacterium *Thermotoga maritima*. These chimeric enzymes retained activity at elevated temperatures (70–90°C) and displayed stability within the 50–70°C range, conditions relevant to industrial biomass conversion. This thermostability reduces enzyme dosage requirements and enhances process efficiency (Gilmore et al., 2020). Furthermore, Kahn et al. (2019) recently developed a hyperthermostable DC system, active at 75°C. Enzymes from *Caldicellulosiruptor bescii*, a cellulolytic hyperthermophilic bacterium, were adapted to the cellulosomal mode by attaching dockerins that matched the thermostable cohesins within a chimeric scaffoldin. Three cohesin-dockerin pairs from three different thermophiles (*C. thermocellum, C. clariflavum* and hyperthermophilic archaeon *Archaeoglobus fulgidus*) were tested and remained stable at 75°C for 72 h. The resulting cellulosome complex showed superior enzymatic activity on microcrystalline cellulose at 75°C, outperforming *C. thermocellum*-based DC and the native *C. thermocellum* cellulosome (Kahn et al., 2019; Stern et al., 2015). Glycosylation of cellulosomal components has also been shown to improve stability and efficiency in cellulose hydrolysis. Inclusion of the glycosylated components yielded an active cellulosome system that exhibited long-term stability at higher temperatures and significantly improved activity compared to the enzymatic components alone (Kahn et al., 2020). These properties are particularly desirable for industrial biomass degradation, where stability and efficiency under harsh process conditions are essential.

Further taking advantage of the modularity of DCs, Vanderstraeten et al. (2022b) successfully reconstituted the free xyloglucan degradation system from *Cellvibrio japonicus* into a cellulosomal format. They engineered and purified constructs combining four catalytic domains appended with dockerin modules from diverse origins and assembled these onto a tetravalent scaffoldin. The resulting “xyloglucanosome” efficiently hydrolyzed xyloglucan into oligosaccharides, galactose, xylose, and glucose, demonstrating the functional integration of complex enzyme systems into DCs (Vanderstraeten et al., 2022b).

When designing novel docking enzymes, the CBM domain of the selected multimodular enzyme is often replaced with a dockerin. This strategy preserves the construct’s natural order of the modules, and, consequently, the optimal catalytic performance of the enzymatic module (Vanderstraeten et al., 2022a). For single-module enzymes, the optimal dockerin position must be determined empirically, as it is difficult to predict. In the native proteins, dockerins are usually positioned C-terminally from enzymatic domain. Nevertheless, they can also be located N-terminally (Mondal et al., 2021; Raghothama et al., 2001) or even internally, between the two enzymatic modules (Grépinet et al., 1988). In DCs, dockerins were found to be functional when placed either N- or C- terminally (Caspi et al., 2009; Caspi et al., 2008; Vanderstraeten et al., 2022a).

The discovery of novel catalytic modules continues to expand the cellulosomal toolbox. For instance, the endoglucanase Rf GH16_21 from *R. flavefaciens* was found to possess a N-terminal dockerin module and three tandem GH16 domains. The enzyme was shown to exhibit notable thermostability and exclusively *β*-1,3–1,4-endoglucanase activity. This activity is likely essential for coordinating the *R. flavefaciens* cellulosome’s degradation of β-1,3–1,4-glucans (Ahmed et al., 2024; Mondal et al., 2021). Similarly, a recent proteomic analysis of the (Pseudo)*Bacteroides cellulosolvens* secretome revealed expression of 24 scaffoldins and 166 dockerin-bearing components (predominantly enzymes), as well as free enzymatic subunits (Zhivin-Nissan et al., 2019), highlighting a rich resource of modular parts for biotechnological applications.

Taken together, these advances show that DCs provide a modular and scalable platform for industrial biomass conversion. Their adaptability—through thermostable, glycosylated, and chimeric components—supports efficient performance under demanding conditions. Combined with improved recombinant expression and assembly methods, these features enable their practical application in large-scale bioprocessing.

## 7 Regulation and adaptive plasticity of cellulosomal activity in response to substrate availability and environmental conditions

Cellulosomes exhibit a remarkable ability to adapt their composition in response to environmental conditions. Carbon sources, pH, temperature, ionic strength, chemical compounds (like inhibitors or enhancers) and chemical or biological pretreatment of the substrate can all affect their activity (reviewed in Wang et al., 2019 and Datta, 2024).

**Figure 4:**
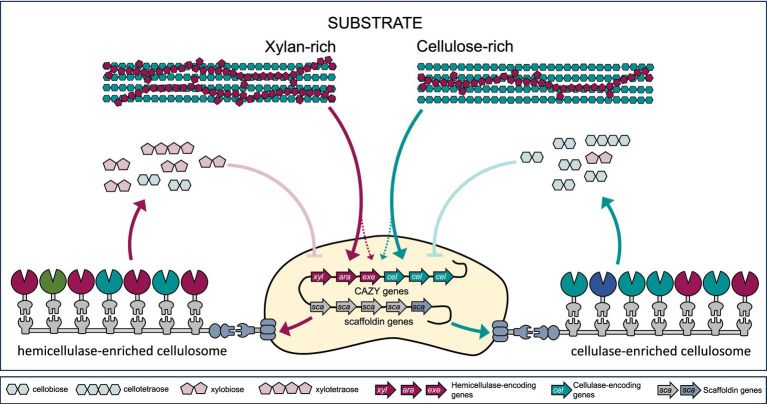
Substrate-mediated regulation of cellulosomal gene expression and complex composition. Adaptive remodeling of cellulosome composition and activity is driven by key regulatory mechanisms, including differential gene expression, enzyme exchangeability, and feedback inhibition. Substrate sensing leads to differential expression of hemicellulase (*xyl, ara, exe*) genes (e.g., endo-β-1,4-xylanase, *α*-L-arabinofuranosidase, β-xylosidase, acetyl xylan esterase, feruloyl esterase), cellulase (*cel*) genes (endo- and exoglucanases), and scaffoldin (*sca*) genes. On the other hand, accumulation of higher concentration of the degradation products can lead to feedback inhibition and decreased complex activity.

The composition and activity of cellulosomes can vary, allowing microorganisms to assemble different enzyme complexes depending on the available carbon source (Figure 4). Upregulation of genes encoding CAZymes, cell motility, chemotaxis, quorum sensing, and some GH regulation was recently detected in a study on *C. thermocellum* B8 during growth on sugarcane bagasse and straw versus purified microcrystalline cellulose (de Camargo et al., 2023). Differential expression of gene clusters targeting different carbohydrate sources, i.e., cellulose or hemicellulose, has also been observed in other bacteria, for example *C. cellulolyticum* (Blouzard et al., 2010; Raman et al., 2009).

Differential composition of catalytic and structural subunits was also detected within cellulosome complexes isolated from *C. thermocellum* grown on different carbon sources. Microcrystalline cellulose- and glucose-derived cellulosome samples exhibited higher endoglucanase-to-exoglucanase ratios and greater catalytic subunit-per-scaffoldin ratios than those derived from lignocellulose (Yoav et al., 2017). A proteome-wide study investigating *B. cellulosolvens* cellulosomes revealed distinct cellulolytic enzyme profiles depending on the carbon source. When grown on microcrystalline cellulose, the bacteria produced cellulosomes displaying the highest expression of the structural and enzymatic subunits. These cellulosomes also displayed the highest degradation activity compared to the ones derived from the bacteria grown on four other cellulosic and hemicellulosic substrates (Zhivin-Nissan et al., 2019). Furthermore, research on *R. cellulolyticum* has revealed that the enzymatic subunits of cellulosomes can readily be exchanged even after assembly, indicating dynamics and flexibility. The strength of dockerin-cohesin interaction is based on specific residues in the dockerin sequence (described in more detail in chapter 2), which can either lock the enzymes in place or allow for reversible binding. This adaptability allows cellulosomes to be refunctionalized over time, enabling them to adjust their catalytic capabilities as needed, a feature that highlights their potential for dynamic environmental response (Borne et al., 2020).

Feedback inhibition in cellulosomal activity occurs when the accumulated end products, such as cellobiose or glucose. These products inhibit the activity of cellulases and other enzymes within the cellulosome, thereby reducing biomass degradation efficiency. Wild-type cellulosomes and their producing strains often fall short of industrial requirements due to such inhibition (Lamote et al., 2023). To address this challenge, strains like *C. thermocellum* DSM1313 have been genetically modified to express extracellular β-glucosidase, which helps to hydrolyze cellobiose and alleviate feedback inhibition (Qi et al., 2021; Zhang et al., 2017). Despite these advancements, challenges remain. For example, the key exoglucanase Cel48S is known to be inhibited by soluble xylan present in lignocellulosic hydrolysates, indicating the importance of timely xylan removal during saccharification (Chen et al., 2022). Strategies to overcome feedback inhibition include engineering enzymes with reduced sensitivity to end products (Atreya et al., 2016), introducing alternative pathways for product removal, or optimizing reaction conditions to minimize product accumulation (Qi et al., 2021).

The modular architecture of cellulosomes, the functional exchangeability of their enzymatic components, and the carbon source–responsive regulation of cellulosomal genes together provide a strong basis for engineering dynamic and environmentally adaptive systems. Using an integrative strategy that leverages structural biology, protein engineering, computational and synthetic biology, cellulosomes can be engineered to dynamically adapt their composition in response to environmental cues such as pH, temperature, substrate type, mechanical stress or inhibitors. A key strategy involves the engineering of cohesin–dockerin pairs with enhanced environmental tolerance (Bugada et al., 2025; Duarte et al., 2023; Galera-Prat et al., 2020; Galera-Prat et al., 2018; Kahn et al., 2019; Moraïs et al., 2016; Vera et al., 2021) including dockerins with dual-binding modes that enable reversible enzyme exchange (Duarte et al., 2021; Kahn et al., 2019). Combining cohesin–dockerin pairs from different species allows precise control of enzyme composition and spatial organization (Borne et al., 2013; Vanderstraeten et al., 2022a). Environmental responsiveness can be built in through pH-dependent binding switches, as demonstrated in *C. acetobutylicum* (Yao et al., 2020), while electrostatic tuning offers additional control over dockerin affinity (Chen et al., 2019; Duarte et al., 2023). Scaffoldins can be modularly designed with tunable linker lengths (Bomble et al., 2011; Dorival et al., 2022) and multidockerin domains for conditional and/or sequential enzyme recruitment (Chen et al., 2024; Chen et al., 2019). Although not yet widely realized, design concepts envision cellulosomes with inducible or environmental-sensing modules that could dynamically control assembly (e.g., via riboswitch-linked dockerins or stimulus-responsive cohesins). Adaptation to inhibitors can be engineered by including enzymes that remove inhibitors (Cao et al., 2018; Qi et al., 2021; Zhang et al., 2017)or enzymes with improved tolerance to inhibitors like furfural and 5-HMF (de Andrades et al., 2024). Additionally, CBMs with dual substrate specificity and putative regulatory functions can act as substrate sensors, potentially influencing enzyme targeting and recruitment based on substrate composition (Boraston et al., 2004). At the genetic level, synthetic regulatory elements such as modular promoters, inducible operons, and riboswitches offer strategies for conditional expression of scaffoldins and enzyme components, enabling dynamic adjustment of catalytic activity in response to environmental signals (e.g., nutrient status, pH, inhibitors; see Müller et al., 2025; Topp et al., 2010). Furthermore, computational modeling and high-throughput screening can facilitate the rational design, prediction, and optimization of these complex multienzyme assemblies (Bomble et al., 2011; Bozkurt et al., 2025; Chen M, et al., 2016; Dorival et al., 2022; Slutzki et al., 2015; Vanderstraeten et al., 2022a). Together, these strategies lay the foundation for construction of environmentally responsive, high-performance cellulosomes tailored for sustainable and flexible biomass processing.

## 8 Ultrastructural studies of cellulosome complexes

Cellulosome is heterogenous and dynamic in nature, which is reflected in its overall structure. The complex can assume different forms. Transmission electron microscopy studies revealed that *C. thermocellum* cellulosomes at early stages of growth appear compact, but take on a more relaxed conformation during the later stages of cultivation (Dorival et al., 2022; Mayer et al., 1987; Tatli et al., 2022). Recent research has shown that upon binding to cellulose, cellulosome structure changes to an elongated, even filamentous shape and morphs dynamically at below 1 min time scale according to requirements of the substrate surface (Eibinger et al., 2020). Similarly, multiple conformations were observed in solution as revealed by small angle X-ray scattering (SAXS) and molecular modeling analyses. It was observed that compact forms maintain structural integrity, while extended forms stabilize interactions with solid substrates (Dorival et al., 2022). To date, no crystallographic structure of an entire cellulosome has been successfully solved; cellulosomal unstructured linkers, scaffoldin flexibility, enzyme heterogeneity, and their glycosylation in most species and the dual mode of binding are probably the reasons why. Thus, researchers must resort to alternative methods and complementary approaches to understand the structural basis for their high efficiency.

Early microscopic studies, SAXS and cryo-electron microscopy (cryo-EM) have been used to study cellulosomal components under near-*in vivo* conditions. These studies revealed the flexibility of cellulosomes to adopt tight or loose conformations (Mayer et al., 1987; Smith and Bayer, 2013). Using a “dissect and build” approach, researchers reconstituted 75% of the scaffoldin CipA from *C. thermocellum*. They found that it adopts compact yet flexible conformations with catalytic domains projecting in alternating directions (Bule et al., 2018a; Currie et al., 2012; García-Alvarez et al., 2011; Smith and Bayer, 2013). These findings highlight an efficient spatial organization within the cellulosome, balancing compactness and flexibility. Interestingly, SAXS and biochemical studies indicated that length and composition of inter-cohesins linkers have limited or no impact on the synergy and activity of cellulosomal cellulases (Molinier et al., 2011). It was also observed that loading scaffoldins with enzymes influences the flexibility of linker regions. Specifically, the more enzymes added, the more compact the structure becomes (Dorival et al., 2022).

**Figure 5:**
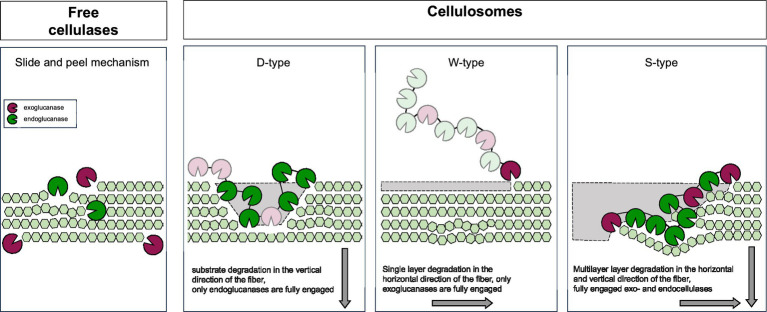
Movement of non-complexed cellulases versus cellulosomes on cellulose microfibrils. Free non-complexed cellulases penetrate the cellulose substrate and move via “slide and peel” mechanism. In contrast, cellulosomes operate in a distinct “sit-and-dig” manner. Functioning as molecular machines, cellulosomes degrade cellulose fibrils through three modes: D-type, W-type, and S-type. The engagement of exo- (magenta) and endoglucanases (green) is indicated by filled circles, and the extent of cellulose removal (gray) reflects the contribution of each mode. The S-type shows full subunit engagement and bidirectional fibril degradation (gray arrows). Adapted from Zajki-Zechmeister et al. (2024; CC BY 4.0).

Real-time movement of the *C. thermocellum* cellulosome complex on a cellulosome fiber (Figure 5) was investigated using atomic force microscopy (AFM) in two recent studies. In the first one, imaging of dynamic structural changes of the cellulosomes interacting with its crystalline cellulose substrate revealed that cellulosomes use a distinct, microfibril cutting and shortening mechanism, a “sit-and-dig” mode, to degrade crystalline cellulose (Eibinger et al., 2020). This is in contrast to a “slide-and-peel” mode of free cellulases (surface ablation; Eibinger et al., 2020; Eibinger et al., 2017; Igarashi et al., 2011; Payne et al., 2015; Resch et al., 2013). These studies also provide a mechanistic explanation for cooperativity observed between cellulosomes and cellulases in cellulose degradation (Eibinger et al., 2020; Resch et al., 2014; Resch et al., 2013). They also explain why in a complex biomass like the plant cell wall, the relatively small cellulases can penetrate into the fibrillar matrix of cell walls, whereas cellulosomes remain primarily on the surface due to their large size (Ding et al., 2012). In the second AFM study, it was discovered that on rough cellulose surfaces, *C. thermocellum* cellulosomes exhibit random conformational changes, accommodating the local nanomorphologies during vertical substrate degradation (D-type). On smooth surfaces, directional movement (W- and S-type moving) depended on the mechanochemical coupling of catalysis and motion via processive exocellulase Cel48S. Both W- and S-type moving cellulosomes functioned as molecular motors using a Brownian ratchet mechanism, in which random motions were directed by Cel48S-mediated cellulose hydrolysis. The “burnt bridge” model explains this movement: as the cellulosome advances, it degrades its track, preventing backward diffusion. S-type cellulosomes achieved multilayer degradation through synergy between Cel48S exocellulases and endocellulases in their aligned-elongated conformation. In contrast, W-type cellulosomes degraded single layers due to inefficient endo-exo synergy, likely caused by the inaccessible local nanostructure of crystalline cellulose (Zajki-Zechmeister et al., 2024).

**Figure 6:**
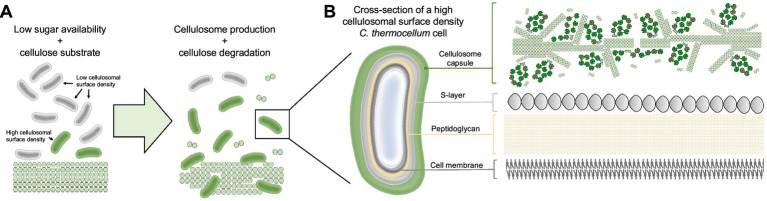
Phenotypic and ultrastructural adaptations of *C. thermocellum* during cellulose degradation. **(A)** *C. thermocellum* cell population displays dynamic phenotypic heterogeneity regulated by sugar availability. Gray and green rods represent cells with low and high density of surface associated cellulosomes, respectively. Under low-sugar conditions and upon cellulose exposure, cellulosome expression increases, expanding the high-density population to enhance cellulose degradation. As sugar accumulates, expression is downregulated, restoring the low-density majority. **(B)** Cross-section of an high-cellulosome density phenotype *C. thermocellum* cell showing the cell membrane, peptidoglycan layer, S-layer and surrounding cellulosome capsule. Cellulosomes are engaged with cellulose fibers, and when in interaction, the compact fibrillar structure is locally disrupted, and the fiber degraded. Adapted from Tatli et al. (2022; CC0 1.0).

Recent nanoscale structural investigations of intact *C. thermocellum* cells provided critical insight into the organization and regulation of cellulosomes at near-physiological conditions (Tatli et al., 2022). Using cryo-EM, cryo-ET, and confocal immunofluorescence, the study found that cellulosomes form a distinct, uniform layer surrounding the bacterial cell at constant distance (~64 nm) from the cell wall (Figure 6). This “cellulosome capsule” physically encases and engages cellulose-degradation intermediates and likely provides proximity between the cellulosomal enzymatic machinery and the substrate. Importantly, the study also uncovered significant phenotypic heterogeneity within the *C. thermocelum* cell population in terms of cellulosome density. Two distinct phenotypes were identified within the same environment and under the same conditions: cells with high-density and cells with low-density cellulosome coverage (Figure 6). Under the stationary phase conditions, the majority of the cells exhibited low-density phenotype, while upon transition into a cellulose-only environment, the proportion of high-density cells markedly increased. These findings reflect the dynamic response linked to the soluble sugar availability. Furthermore, in the presence of soluble sugars (5 mM glucose or cellobiose), the expression of key cellulosomal components, such as the exocellulase Cel48S, was downregulated, suggesting a feedback inhibition of cellulosome production by products from the cellulose-degradation process. Even further, the presence of both phenotypes in the population and changes in their ratio also suggest a division of labor within the bacterial population. Herein, a bet-hedging strategy of costly cellulosome expression even when soluble sugars are available keeps the population prepared for future cellulose-rich environments (Tatli et al., 2022).

Altogether, these findings highlight how the cellulosomal structural flexibility enables functional adaptability. Compact conformations support enzyme clustering and structural stability, while extended or motile states optimize substrate engagement, spatial exploration, and degradation efficiency. This adaptability is essential for navigating heterogeneous substrates and fluctuating environmental conditions.

## 9 Limitations and challenges of cellulosome-based strategies in biorefinery applications

The integration of natural and designer cellulosomes into biorefinery platforms holds significant promise for efficient lignocellulosic biomass conversion, yet their industrial deployment faces several critical bottlenecks. Firstly, despite the cellulosome’s inherently synergistic architecture, effective hydrolysis still relies on costly pretreatment methods to enhance substrate accessibility, often negating the economic advantage of consolidated bioprocessing strategies (Liu et al., 2019; Wang et al., 2019; Yu et al., 2020).

Natural cellulosomes, such as those produced by *C. thermocellum*, also suffer from compositional limitations. For example, the absence of *β*-glucosidase leads to cellobiose accumulation and feedback inhibition (Hirano et al., 2019; Kruus et al., 1995). DCs attempt to overcome such constraints through synthetic scaffolding of multiple enzymes, but their performance on pretreated biomass frequently remains inferior to optimized free-enzyme cocktails, achieving only 33–42% of native cellulosome efficiency (Chundawat et al., 2016; Gefen et al., 2012; Hirano et al., 2019; Moraïs et al., 2012). Despite proven efficacy of proximity-driven synergy between cellulases and accessory enzymes such as LPMOs, xylanases, laccases, or expansin-like proteins, defining optimal enzyme combinations within the expansive design space remains challenging. Furthermore, the production and assembly of complex multienzyme designer systems are technically demanding, costly, and not yet scalable. The immense combinatorial possibilities of scaffoldin architectures and enzyme modules make high-throughput screening and rational design exceptionally difficult to implement (Lamote et al., 2023; Tsai et al., 2022).

In consolidated bioprocessing strategies contexts, metabolic burden imposed by heterologous enzyme expression in engineered hosts such as *Saccharomyces cerevisiae* often compromises fermentation efficiency (Dong et al., 2020b; Sharma et al., 2022; Song et al., 2025; Tsai et al., 2022). Additionally, host organisms that naturally produce cellulosomes are difficult to genetically manipulate (Olson et al., 2023; Riley et al., 2019) and may lack robust industrial traits (Mazzoli and Olson, 2020). On the biochemical level, trade-offs between (thermo)stability and enzymatic activity (Kahn et al., 2019) as well as steric hindrance during enzyme immobilization (Vazana et al., 2013) limit the practical stability and reusability of cellulosomal systems. Altogether, these barriers, ranging from substrate challenges and enzyme composition to host engineering and process economics, highlight the need for continued advances in synthetic biology, pretreatment innovation, and techno-economic optimization to enable the viable industrial application of cellulosome-based technologies in biorefineries.

## 10 Conclusion and outlook

Despite recent advances highlighting the vast potential of optimized cellulosome design for improved polysaccharide processing, several challenges remain. The dynamic modularity and component heterogeneity of cellulosomes continue to hinder high-resolution structural characterization and mechanistic understanding of their assembly and function in native environments.

One major area of interest is the optimization of CBMs, which are pivotal for substrate targeting and enzyme–substrate proximity. However, challenges persist in improving thermostability and substrate specificity, as well as optimizing the spatial arrangement and functional integration of CBMs with catalytic domains. Furthermore, the multivalency and cooperativity of CBMs in the context of native or engineered cellulosomes remain incompletely understood, limiting the rational design of more efficient cellulolytic systems.

Equally important is the need to better understand cohesin–dockerin interactions, particularly dual-binding modes that underlie the dynamic adaptability of cellulosomes. These interactions remain poorly understood, especially in wild-type systems. In addition, less-studied type III cohesin-dockerin interactions, together with advances in genomics, structural biology, and bioinformatics, offer opportunities to uncover new functions and engineer next-generation cellulosomal assemblies. Notably, some cohesin-dockerin systems have even been proposed to function beyond classical polysaccharide degradation, potentially contributing to extracellular protein or lipid breakdown, microbial community interactions, and environmental sensing.

Another key challenge lies in understanding and optimizing cell surface attachment mechanisms. This includes identification of novel anchoring mechanisms beyond classical SLH and dockerin-cohesin mediated interactions, and optimization of anchorage efficiency, robustness, and flexibility, particularly for compatibility with the non-native hosts. Moreover, the dynamic regulation of attachment systems to adapt to changes in the environmental conditions warrants further investigation.

Realizing the full potential of DCs will also require a deeper understanding of enzymatic synergy between cellulases and hemicellulases, the mechanisms of product and intermediate inhibition, and the functional roles of associated enzymes with atypical catalytic activities, such as proteases or redox enzymes, that may contribute to biomass deconstruction.

DCs offer a promising strategy to reduce the environmental footprint and improve the economic viability of lignocellulosic biorefineries. By enhancing catalytic efficiency, DCs lower enzyme usage, reduce energy demand and transport emissions and support local bioeconomies. Realizing their full potential will require a coordinated research roadmap that merges mechanistic insight with rational engineering: (1) applying high-resolution cryo-EM, NMR, and single-molecule techniques (e.g., FRET, optical tweezers) to capture the transient cohesin–dockerin, CBM, and catalytic-domain interactions; (2) mining genomes and metagenomes to uncover novel DC-building blocks; (3) using molecular-dynamics simulations to refine domain architecture, dockerin-cohesin interactions, linker flexibility, substrate binding and surface-display; (4) integrating transcriptomic, proteomic, and metabolomic data in systems-biology models to understand and engineer regulatory mechanisms controlling cellulosome expression and anchoring; and (5) iterating computational design, modular assembly, and high-throughput screening to generate DCs tailored for specific microbial chassis and lignocellulosic substrates. By uniting these experimental and computational tools within a sustainability-driven framework, the field is poised to deliver high-performance, application-specific cellulosomal platforms that can power the next generation of biomass-valorization technologies.

## Footnotes

^1^ http://www.cazy.org/
